# Supplementary material for: Opposing Effects of Prior Infection versus Prior Vaccination on Vaccine Immunogenicity against Influenza A(H3N2) Viruses
Source: Viruses. 2022 Feb 25;14(3):470. doi: 10.3390/v14030470 (PMC8949461; doi:10.3390/v14030470)
Supplement: Supplementary file 1 [file viruses-14-00470-s001.zip › viruses-1371208-supplementary.pdf]

viruses

MDPI

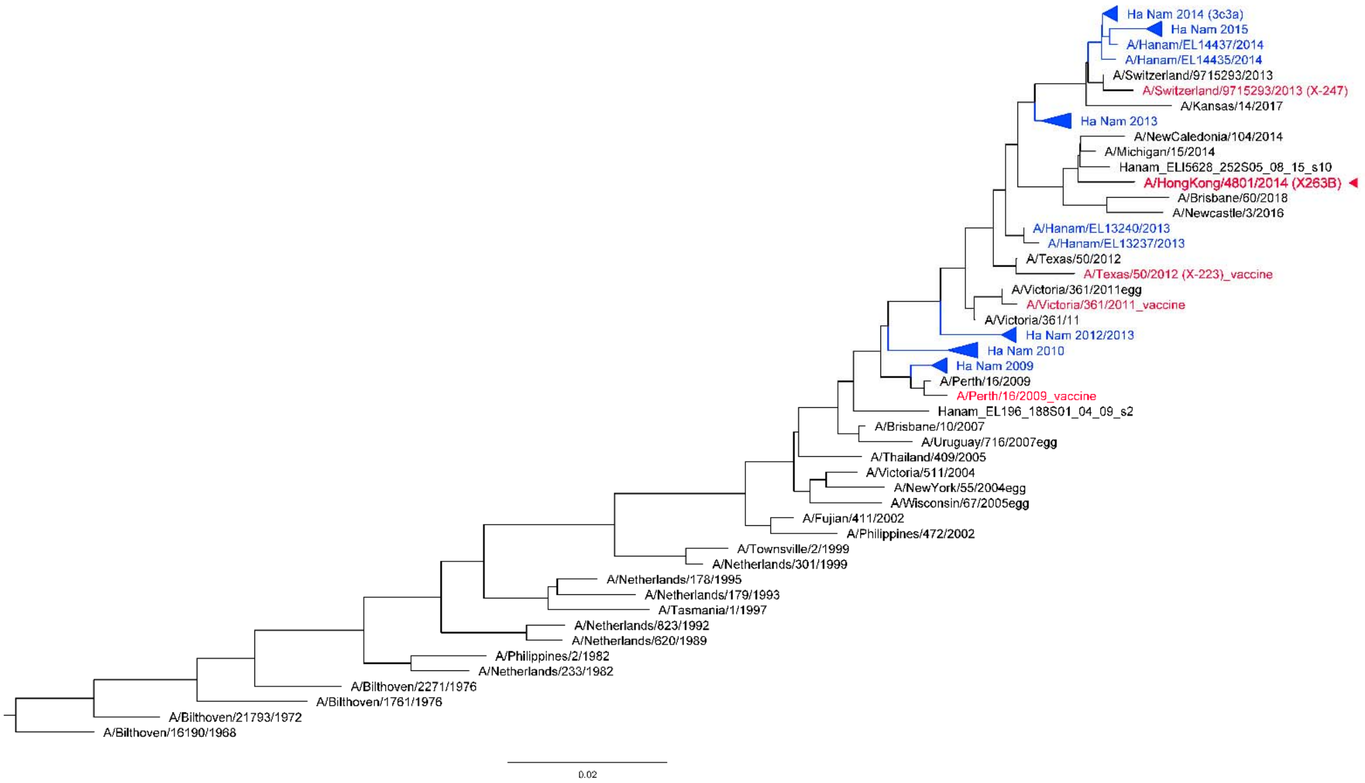

Figure S1. Phylogenetic tree of HA genes of A(H3N2) strains that participants were exposed to by infection or vaccination, and that were used to detect serum antibodies by HI assay. Vaccine strains used between 2011 and 2016 are coloured red. Strains recovered from Ha Nam Cohort Participants between 2009 and 2016 are coloured blue.

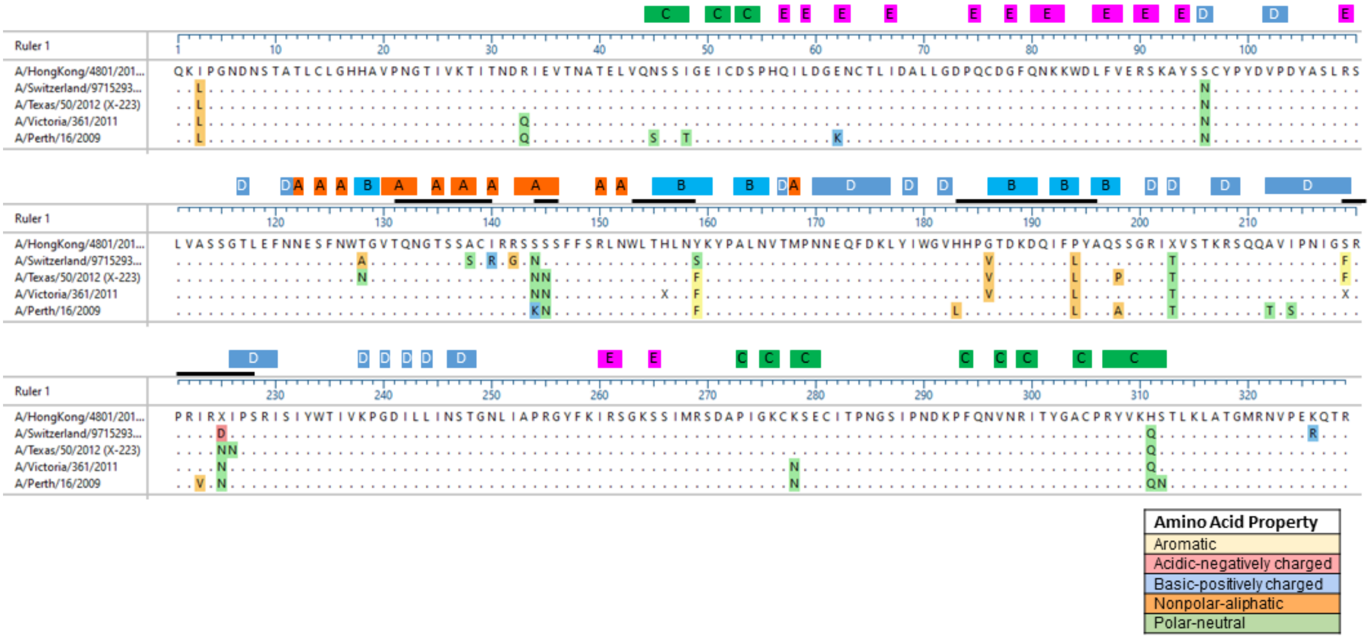

Figure S2. Alignment of HA1 amino acid sequences of A(H3N2) vaccine strains used during the study period (2011-2016). Amino acids used are shown for A/Hong Kong/4801/2014 (egg-grown), and for variant positions of earlier strains. Variant amino acids are coloured according to amino acid properties (legend). Antigenic site positions are indicated and colour-coded above alignments. Receptor binding domain positions are indicates by the black bars above the alignments.

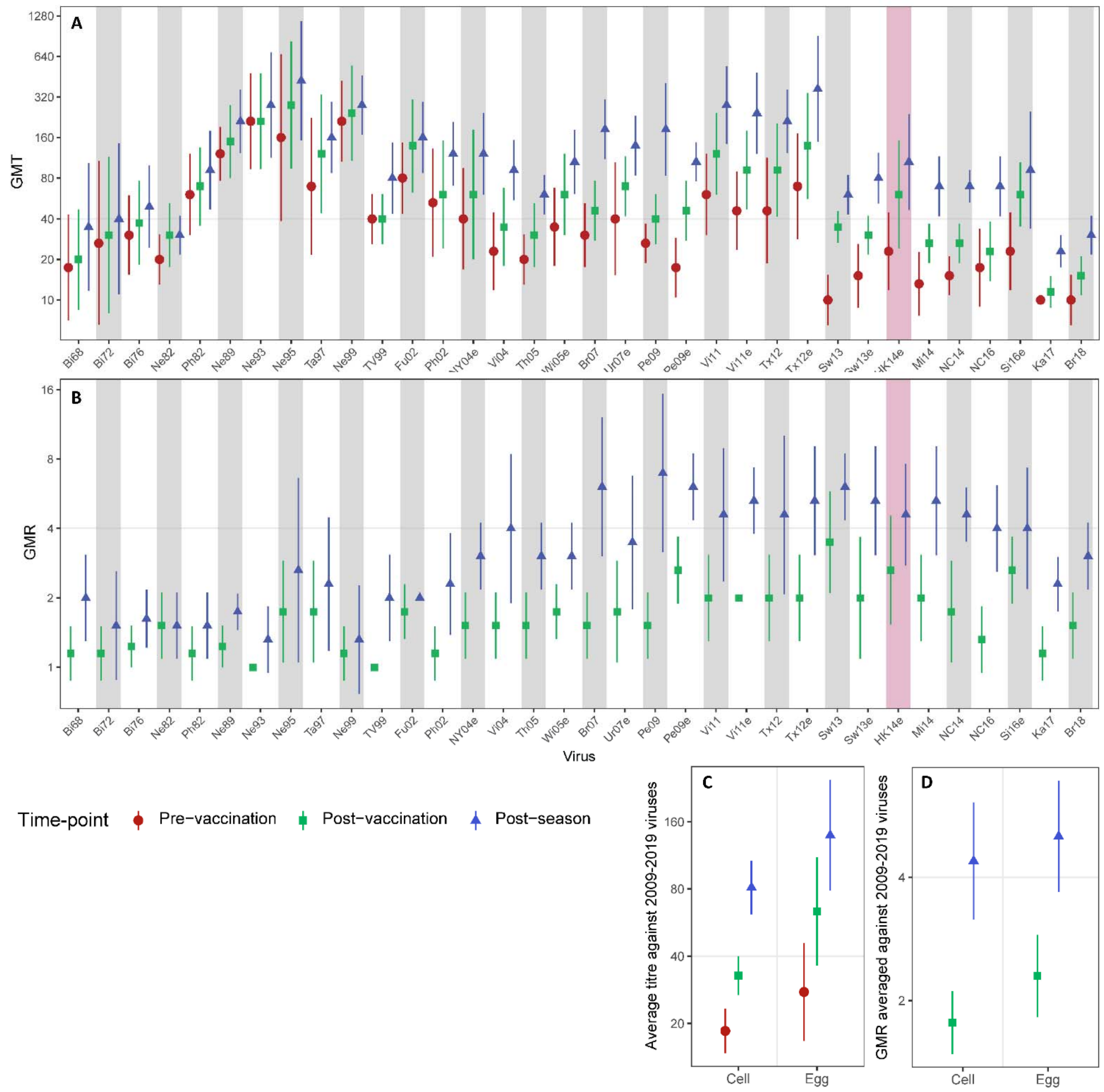

Figure S3. Post-vaccination versus post-season titre rises of HCWs who developed A(H3N2) virus confirmed ILI in the season after vaccination. Results for five HCWs are shown as geometric mean titres (GMT) or ratios (GMR) for individual viruses (**A**,**B**) or groups of viruses circulating since 2009 (**C**,**D**). Error bars are 95% confidence intervals. Post-vaccination sera were collected 20-26 days post-vaccination whereas post-season sera were collected 222-224 days post-vaccination, which was 82-97 days post ILI onset. Infecting strains were A/Newcastle/30/2016-like (NC16) 46. The vaccine strain is indicated by the red shaded panel.
